# Polyethyleneimine-treated polyacrylonitrile membrane hemofilter for critically ill patients receiving anticoagulant-free prolonged intermittent renal replacement therapy: a single-center, prospective, self-controlled pilot study

**DOI:** 10.1186/s12882-017-0627-1

**Published:** 2017-06-30

**Authors:** Tao Su, Qizhuang Jin, Zhongyuan Liu

**Affiliations:** Renal Division, Department of Medicine, Peking University First Hospital, Peking University Institute of Nephrology, Beijing, 100034 China

**Keywords:** Anticoagulant-free, Surface-treated polyacrylonitrile membrane hemofilter, Polyethyleneimine, Prolonged intermittent renal replacement therapy

## Abstract

**Background:**

Critically ill patients requiring renal replacement therapy (RRT) are at risk of disease-related bleeding. Systemic heparinization should be avoided. AN69ST, a polyethyleneimine-treated polyacrylonitrile (AN69) membrane hemofilter, can be primed with heparin, improving its local anticoagulative activity. Prolonged intermittent RRT (PI-RRT) is of shorter duration and cheaper, considered as an alternative to continuous RRT. This study was performed to compare the success rate of anticoagulant-free PI-RRT using AN69ST versus AN69 membrane hemofilter. We also evaluated risk factors for filter clotting.

**Methods:**

This crossover, double-blind, randomized study included patients requiring PI-RRT but at high bleeding risk treated with AN69ST and AN69 hemo filters. The success rate of RRT, filter lifespan and severity of filter clotting were compared between the hemo filters. Factors associated with the filter clotting risk were analyzed with a Cox proportional hazards model.

**Results:**

This study included 60 patients (mean age, 68.1 ± 15.8 years). Thirty-three (55.0%) patients were in the intensive care unit, 34 (56.8%) had disease-related thrombocytopenia, and 14 (23.3%) had local hemorrhagic diseases. The success rate of PI-RRT with the AN69ST and AN69 hemofilter was 51.7% and 50.9%, respectively (*P* > 0.05). The mean PI-RRT duration was 543.1 ± 119.0 min in the completed sessions and 387.3 ± 140.8 min in the prematurely terminated sessions, without significant difference between AN69ST and AN69 hemofilters. Cox regression analysis showed that age (odds ratio [OR], 1.023 per year), platelet count (OR, 1.07 per 10 × 10^9^/L), hemoglobin concentration (OR, 1.035 per 1 g/L), and activated partial thromboplastin time (aPTT; OR, 0.973 per second) were associated with a hemofilter clotting risk. The AN69ST hemofilter lifespan was significantly prolonged averaging an extra 251.7 min in patients with an aPTT of <35.3 s, hemoglobin concentration of >83 g/L and platelet count of <70 × 10^9^/L.

**Conclusions:**

Anticoagulant-free PI-RRT by a heparin-primed AN69ST hemofilter reached a 51.7% success rate. The risk of premature clotting of the extracorporeal circuit remains unsatisfactory. For select patients at high risk of bleeding, the heparin-primed AN69ST hemofilter may be more appropriate for anticoagulation-free PI-RRT.

**Trial registration:**

https://www.clinicaltrials.gov; study number: NCT02355873. Release Date 01/21/2015

## Background

Systemic anticoagulation is necessary in patients undergoing continuous renal replacement therapy (CRRT) to avoid premature termination and low efficacy of blood purification due to clotting of the extracorporeal circuit including hemofilter [1, 2]. Either unfractionated heparin or low-molecular-weight heparin can be injected intravenously to achieve systemic heparinization. However, continuous heparinization exposes patients to an increased risk of bleeding, especially critically ill patients with coagulation disturbances. Management such as saline flushing or pre-dilution mode without heparin has long been considered unsatisfactory [3, 4], and is used to be performed instead of systemic heparinization for patients undergoing maintenance hemodialysis. For patients with acute kidney injury at high risk of bleeding requiring CRRT, the Kidney Disease: Improving Global Outcomes criteria recommend regional citrate anticoagulation (RCA). However, this anticoagulation regimen increases the complexity of the operation and monitoring; places patients at risk of disorders involving calcium, sodium, and acid–base imbalances; and is also more costly because it requires additional nursing care [5, 6]. Modification of the hemofilter to improve its biocompatibility and anticoagulant properties may be another simple and effective approach. The AN69ST hemofilter is a surface-treated polyacrylonitrile (AN69) membrane hemofilter with a polyethyleneimine (PEI) layer, allowing for incorporation of a heparin layer by priming the membrane in a heparin-saline solution before RRT, thereby reducing local thrombogenesis when compared with the original AN69 membrane [7, 8]. Heparin-primed AN69ST membranes have been successfully used in 4-h dialysis and 6-h extracorporeal circulation in previous studies [3, 9–14] and are reportedly more biocompatible with advantages in terms of inflammatory cytokine adsorption [15]. For patients receiving prolonged renal replacement therapy (RRT), evidence regarding the use of a surface-treated membrane hemofilter is scarce. Prolonged intermittent RRT (PI-RRT) is an alternative to CRRT with a shorter duration usually between 6 and 12 h [16, 17]. It is less costly and requires a lower dose of anticoagulants because of the shortened duration.

In this study, we tested the hypothesis that in critically ill patients requiring PI-RRT who were at risk of bleeding and cannot undergo systemic heparinization, performance of the AN69ST hemofilter primed with heparin for anticoagulant-free PI-RRT is more effective than use of the original AN69 hemofilter.

## Methods

### Patients

The inclusion criteria for this study were an age of ≥18 years, requirement for PI-RRT, use of well-functioning double-lumen catheters providing a dialysis blood flow rate of 150 ml/min, and the presence of a bleeding risk contraindicating the use of continuous heparinization during RRT.

Patients undergoing treatment with oral antiplatelet drugs or injectable low-molecular-weight heparin were excluded from this study. The other exclusion criteria were an expected survival time of <72 h, extremely unstable vital signs such as a low blood pressure that was unresponsive to fluid therapy and vasopressors, pregnancy, and heparin allergy.

### Filter membrane and pretreatment before RRT

The AN69ST hemofilter is a PEI-treated AN69 membrane with a surface area of 1.3 m^2^ (Surface-treated Multiflow 100, hollow-fiber 1.3 m^2^; Gambro-Hospal, Meyzieu, France). The second PEI-layer is able to be primed with a third heparin layer in a heparin-saline solution. The AN69 hemofilter is an AN69 membrane with a surface area of 1.3 m^2^ (Multiflow 100, hollow-fiber 1.3 m^2^; Gambro-Hospal).

Both hemofilters were primed with heparin saline (unfractionated heparin, 50 mg/6250 units/L) at 100 ml/min for 10 min in an open-loop circuit. After circulation, the heparin saline was flushed out of the hemofilter and circuit tubes prior to starting PI-RRT.

### Study design

This was an interventional, crossover, double-blind, randomized, self-controlled trial. The study was conducted according to national regulations and was approved by the local ethics committee (https://www.clinicaltrials.gov ID: NCT02355873). All patients provided written informed consent before enrollment.

**Fig. 1:**
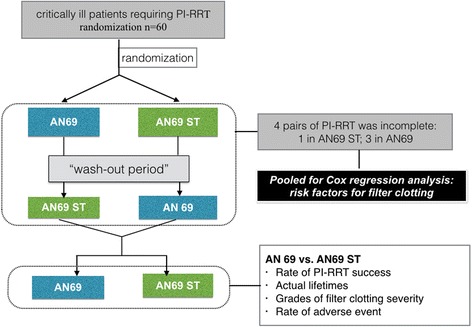
Flowchart of the study

All patients underwent two successive sessions of PI-RRT with an AN69ST and an AN69 hemofilter. The order of the sessions was randomized using a random number table, followed by crossover to the other type of hemofilter. All numbers were placed in envelopes to ensure double-blinding. After a wash-out interval of 10 h, the patients were switched to the second session with the alternative hemofilter. The study flowchart is shown in Fig. 1.

### PI-RRT strategy

The double-lumen catheters were placed into the jugular or femoral vein before PI- RRT. The catheter lock concentration of heparin was 10 mg/L.

All patients underwent continuous venovenous hemofiltration (CVVHF) with a substitution flow rate of 3000 ml/h in mixed-dilution mode (pre-dilution, 2000 ml/h; post-dilution, 1000 ml/h). The blood flow rate was maintained at 180 to 250 ml/min. The ultrafiltration rate was set according to each patient’s scheduled dehydration goal. All parameters were maintained within subgroups of patients who controlled their own hemofiltration protocols. The Prismaflex system (Gambro) was used in the present study.

No anticoagulant was used during any of the PI- RRT procedures. During PI- RRT, occlusion of the hemofilter or venous bubble trap may result in circuit clotting. A clotting event was defined as a transmembrane pressure of >280 mmHg and/or a circuit venous pressure increase of >300 mmHg without a vascular access problem or blood clotting of the venous bubble trap. The number of PI-RRT sessions that were stopped early was recorded.

The hemofilters were inspected at the end of each PI-RRT session. Filter clotting was graded as 0 (normal), 1 (blood stripes in <5% of the fibers), 2 (blood stripes in 5%–50% of the fibers), or 3 (blood stripes in >50% of the fibers). Adverse events and the actual duration of each session were also recorded.

### Study outcomes and statistical methods

The sample size was calculated according to the following hypotheses. Because no previous data have been reported, we conducted a preliminary experiment in four patients. The expected success rate of PI-RRT sessions with circuit clotting was 50% for both filters. The median difference in the duration of PI-RRT between the AN69ST and AN69 membrane hemofilters was 110 min. Thus, we estimated that in the best scenario of PI-RRT without heparin, the expected extension of the duration of PI- RRT by the AN69ST hemofilter was 60 min. The square root of within mean square error was set at between 100 and 130 min. The two-sided statistically significant level and study power were 5% and 80%, respectively. We used the “tests for the two means in a 2 by 2 cross-over design” procedure in the PASS software (version11.0, NCSS, LLC. Kaysville, Utah, USA) to calculate the sample size. Thus, 46–76 subjects were needed based on the above mentioned parameters. We ultimately set the sample size at 60 subjects. And the number of sessions to be performed was 120.

We evaluated the actual lifetimes of the AN69ST and AN69 hemofilters during heparin-free PI-RRT. Continuous variables are expressed as mean ± standard deviation. Differences of lifetimes between the two hemofilters were compared using the self-matching Student’s t-test. The success rates of the AN69 and AN69ST filters were compared, as were the filter clotting severity grades and adverse event rates. Differences in serum marker concentrations before and after PI-RRT using the AN69ST and AN69 filters were also compared by paired t-tests. The patients were pooled for Cox proportional hazards regression analysis to determine the risk factors associated with the PI-RRT survival status. Covariates included age, sex, hemoglobin concentration, platelet count, and parameters associated with the coagulation process, including the prothrombin time (PT) and activated partial thromboplastin time (aPTT). Hazard ratios and 95% confidence intervals were also calculated. A two-tailed *P* value of <0.05 was considered significant. All analyses were performed using SPSS software (version 20.0; IBM Corp., Armonk, NY).

## Results

Patients were recruited from February 2014 to October 2015; ultimately, 60 patients were included and provided informed consent. All were critically ill with acute kidney injury (AKI), 38 (63.3%) cases having a pre-existing chronic kidney injury, and 33 (55%) of the patients were recruited from the intensive care unit. The patients had life-threatening conditions including severe pneumonia; acute coronary syndrome; antineutrophil cytoplasmic antibody positive vasculitis; systemic lupus erythematosus; malignancies involving the intestine, rectum, pancreas, bladder, ureter, or pelvis; and multiple myeloma. Eighteen (30.0%) patients were receiving artificial respiratory support. Forty (66.7%) patients had acute kidney injury, and others were diagnosed with aggravation of pre-existing chronic kidney disease. Altogether, 40 men and 20 women were enrolled, with a mean age of 68.1 ± 15.8 years. Of the 60 patients, 34 (56.8%) had disease-related thrombocytopenia; 14 (23.3%) had hemorrhagic diseases involving a local area, including gastrointestinal bleeding, cerebral hemorrhage, or diffuse alveoli hemorrhage; and others were recovering from surgery.

Sixty pairs of PI-RRT sessions were scheduled to continue for 8 to 12 h in each patient, mainly for relief of water overload and endotoxin accumulation. The actual duration of each session was dependent upon the individual patient’s scheduled dehydration goal and adjusted to ensure comparative values within the AN69ST and AN69 subgroups. Four pairs of intended PI-RRT sessions were incomplete because of sudden worsening of the patient condition (the AN69ST session was not performed in one patient, and the AN69 session was not performed in three patients). The success rate of the heparin-free PI-RRT sessions with the AN69ST and AN69 hemofilter was 51.7% and 50.9% respectively (*P* > 0.05). The AN69 subgroup had a 3.5% occurrence rate of reported adverse events. The PI-RRT sessions were completed as scheduled after a duration of 543.1 ± 119.0 min. The remaining sessions continued for an average 387.3 ± 140.8 min and were terminated early. There were no significant differences in the actual lifespans of the AN69 and AN69ST hemofilters, in both the prematurely terminated (404.8 ± 142.0 vs. 366.9 ± 140.0 min, respectively; *P* > 0.05) and successfully completed PI-RRT sessions (540.2 ± 117.8 vs. 546.2 ± 122.1 min, respectively; *P* > 0.05).

In the AN69ST group, grade 0, 1, 2, and 3 filter clotting occurred in 10.3%, 17.2%, 12.1%, and 60.3% of patients, respectively. No significant differences were found when compared with the AN69 group (5.5%, 14.0%, 17.5%, and 63.2%, respectively). There was a low incidence of venous bubble trap occlusion in both groups (AN69ST, 6.9%; AN69, 5.3%). Following the 56 prematurely terminated sessions, most filters (*n* = 52, 92.9%) had grade 3 filter clotting. Only one session had to be stopped because of venous bubble trap occlusion with grade 2 hemofilter clotting. These details are shown in Tables 1 and 2.

**Table 1:** Comparison of early termination and on-schedule completion of PI- RRT

	RRT Early terminated *n* = 60	RRT Completed on schedule *n* = 54	*P* value
RRT treatment time (min)	387.3 ± 140.8	543.1 ± 119.0	<0.001
UF volume (kg)	1.7 ± 1.1	2.3 ± 1.0	0.008
Age (year)	70.9 ± 13.4	66.1 ± 16.9	0.101
pre PLT (× 10 ^9^ /L)	138.4 ± 90.9	119.4 ± 92.5	0.285
pre Hb (g/L)	89.0 ± 14.3	82.0 ± 14.6	0.013
pre WBC (× 10 ^9^ /L)	11.3 ± 10.5	9.6 ± 4.7	0.268
pre PT (s)	13.1 ± 2.0	16.5 ± 20.3	0.205
pre aPTT (s)	35.2 ± 10.6	41.5 ± 21.5	0.065
pre Alb (g/L)	34.7 ± 43.3	31.6 ± 6.1	0.609
pre SCr (μmol/L)	361.6 ± 218.3	360.3 ± 248.0	0.978
pre BUN (mmol/L)	27.1 ± 45.0	20.5 ± 8.63	0.275
Change of SCr %	27.7 ± 16.6	34.4 ± 12.1	0.074
URR %	28.0 ± 13.0	32.6 ± 11.1	0.036

**Table 2:** Comparison of early termination and on-schedule completion of RRT in the AN69 and AN69ST groups

	RRT completed on schedule			RRT early terminated		
RRT time (min)	543.1 ± 118.9			387.3 ± 140.8		
	AN69	AN69 ST	*P* value	AN69	AN69 ST	*P* value
	567.1 ± 119.9	548.3 ± 128.2	NS	366.9 ± 139.7	404.8 ± 142.0	NS
BFR ml/min	205.2 ± 16.9	202.4 ± 10.9	NS	206.5 ± 22.9	206.2 ± 22.8	NS
UF ml	2161.9 ± 908.0	2142.9 ± 1073.6	NS	1795.8 ± 1204.1	1751.8 ± 948.7	NS
Before RRT						
PLT X10^9	115.9 ± 83.0	108.7 ± 71.7	NS	145.5 ± 109.2	132.4 ± 73.5	NS
Hb g/L	83.1 ± 10.4	81.7 ± 14.3	NS	90.8 ± 13.6	87.4 ± 15.0	NS
WBC X10^9	9.37 ± 4.58	9.46 ± 4.81	NS	11.8 ± 13.8	10.9 ± 6.8	NS
PT s	13.71 ± 4.91	13.25 ± 3.65	NS	13.2 ± 1.7	13.0 ± 2.2	NS
APTT s	42.41 ± 20.80	42.71 ± 30.0	NS	36.2 ± 9.9	34.4 ± 11.2	NS
ALB g/L	31.3 ± 4.16	32.0 ± 4.1	NS	28.4 ± 5.9	28.5 ± 4.3	NS
SCr umol/L	356.1 ± 260.5	402.0 ± 288.8	NS	338.9 ± 175.0	381.0 ± 251.3	NS
BUN mmol/L	19.24 ± 9.00	21.70 ± 7.53	NS	20.6 ± 8.7	21.2 ± 10.0	NS
After RRT						
PLT	93.7 ± 57.7	100.6 ± 67.7	NS	152.6 ± 106.0	125.2 ± 68.1	NS
Hb	84.7 ± 13.4	83.3 ± 14.8	NS	89.9 ± 15.1	86.8 ± 17.9	NS
WBC X10^9	8.94 ± 5.16	8.22 ± 4.40	NS	8.81 ± 4.62	10.3 ± 5.4	NS
PT	13.63 ± 4.22	13.00 ± 2.86	NS	13.0 ± 1.8	13.4 ± 4.1	NS
APTT	48.5 ± 44.01	34.78 ± 6.16	NS	33.7 ± 9.3	30.1 ± 5.1	NS
ALB	33.8 ± 4.7	33.9 ± 4.5	NS	28.2 ± 5.6	29.4 ± 5.5	NS
SCr	241.7 ± 186.4	252.5 ± 199.4	NS	260.1 ± 151.8	279.2 ± 179.8	NS
BUN	12.5 ± 5.8	21.1 ± 31.6	NS	15.2 ± 7.1	15.9 ± 7.8	NS
clotting grades of filters 0/1/2/3	2/7/8/12	5/10/5/10		0/0/1/23	0/2/2/23	
Vein well cloting 0/1	29/1	29/1		22/2	24/3	

The PI-RRT sessions were regrouped according to whether the sessions were terminated early or completed on schedule. The early termination group showed a trend toward a higher hemoglobin concentration (82.0 vs. 89.0 g/L, *P* = 0.013) and platelet count (119.4 ± 92.5 vs. 138.4 ± 90.9 × 10^9^/L, *P* = 0.285) and a lower PT (13.1 vs. 16.5 s, *P* = 0.205) and aPTT (35.2 vs. 41.5 s, *P* = 0.065) when compared with sessions that were completed on schedule. Premature termination resulted not only in inadequate dehydration (average 0.6 kg less) but also in lower and unsatisfactory clearance of urea (28.0%). Cox regression analysis showed that age (odds ratio [OR], 1.023 per year), platelet count (OR, 1.07 per 10 × 10^9^/L), hemoglobin concentration (OR, 1.035 per 1 g/L), and aPTT (OR, 0.973 per second) were associated with a hemofilter clotting risk. Thus, the platelet count is the strongest risk factor associated with the filter lifespan.

**Fig. 2:**
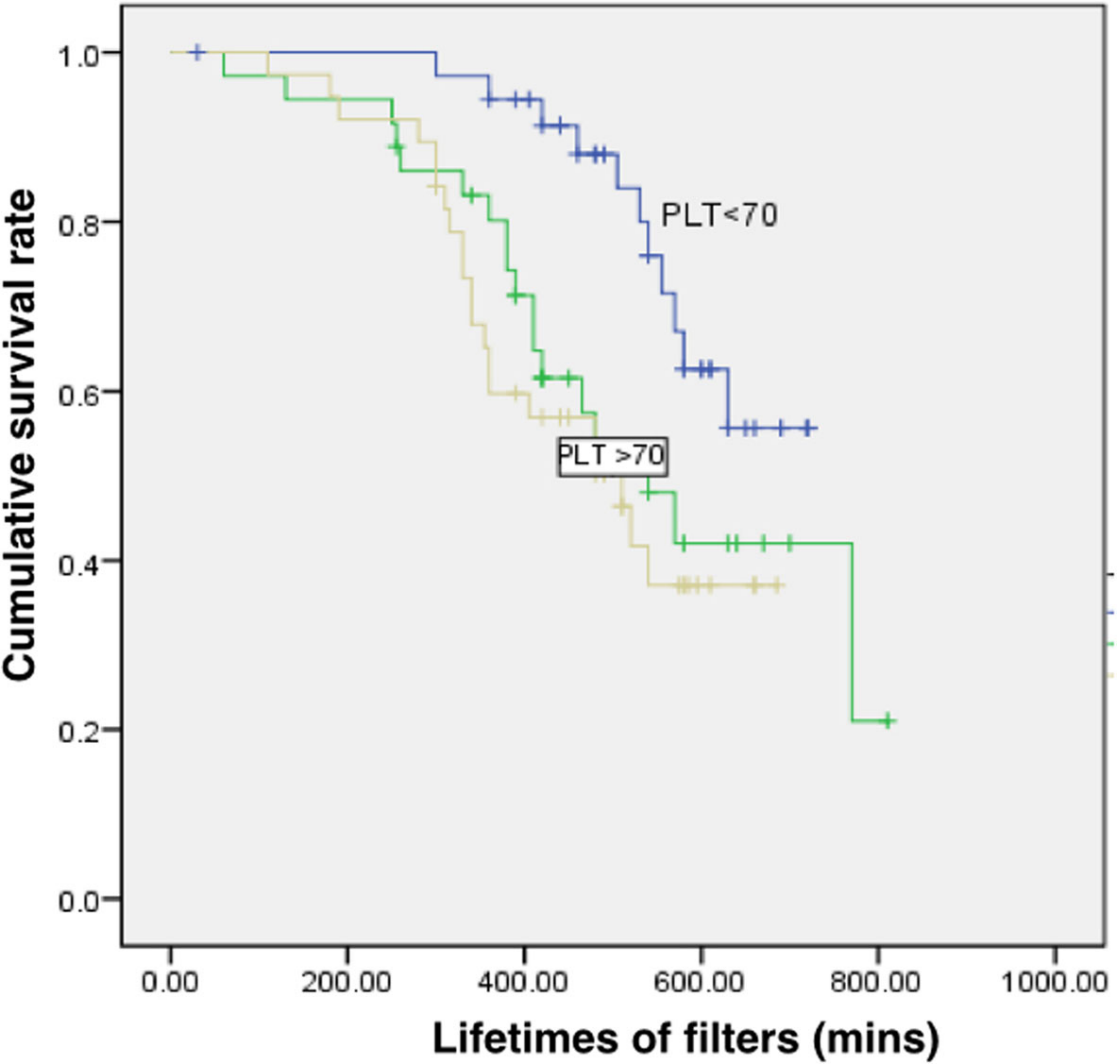
Risk of early termination of prolonged intermittent renal replacement therapy according to platelet level. Patients were divided into three subgroups according to platelet levels of <70, 70 to 150, and >150 × 10^9^/L

The mean platelet count before PI-RRT was 126.4 ± 91.4 × 10^9^/L. The patients had a wide range of platelet counts from 6 to 538 × 10^9^/L. Thrombocytopenia was mainly caused by hematologic malignancies such as myeloma and disease-related platelet consumption. About one-third of patients (*n* = 21, 35.1%) had a very low platelet count of <70 × 10^9^/L. Three patients (5%) had disease-related thrombocytosis. In a further analysis, the patients were equally divided into three groups according to their pre-RRT platelet count. The Kaplan–Meier survival curves of the hemofilter lifespan in these groups are shown in Fig. 2. The hemofilter lifespan was significantly prolonged in patients with a platelet count of <70 × 10^9^/L (*P* < 0.05). The pre-RRT hemoglobin concentration was 85.0 ± 14.8 g/L (median, 83 g/L), but there was no significant difference between patients with a platelet count of >70 and <70 × 10^9^/L (85.1 ± 13.7 vs. 85.8 ± 16.6 g/L, respectively; *P* = 0.804), similar results were found for the aPTT prior to PI-RRT (37.5 ± 13.1 vs. 41.2 ± 23.8 s, respectively; *P* = 0.291). Roughly half of patients had an aPTT longer than the local reference value of 35.3 s. As shown in Table 3, subsequent analysis revealed a significantly prolonged AN69ST hemofilter lifespan in patients with a normal aPTT of <35.3 s, hemoglobin concentration of >83 g/L, and platelet count of <70 × 10^9^/L (extension of 251.7 min). We further compared the pre-RRT aPTT level with that of post-RRT by paired t-test. No significant difference was observed (38.8 ± 17.4 vs. 36.5 ± 20.3 s, *p* > 0.05). It is similar with that of PT, platelet count and hemoglobin.

**Table 3:** Difference in lifespans in min of AN69ST and AN69 membrane hemofilters

	aPTT < 35.3 s				aPTT > 35.3 s			
	Hb > 83 g/L		Hb < 83 g/L		Hb > 83 g/L		Hb < 83 g/L	
	PLT < 70	>70	<70	>70	PLT < 70	>70	<70	>70
	*N* = 6	*N* = 10	*N* = 5	*N* = 8	*N* = 5	*N* = 5	*N* = 3	*N* = 8
AN69 ST	605 ± 103.3	435.5 ± 135.4	538.0 ± 119.5	350.0 ± 101.5	582.0 ± 110.1	404.0 ± 159.5	423.3 ± 136.5	464.4 ± 127.4
AN69	353.3 ± 171.1	441.5 ± 161.7	572.0 ± 108.0	363.8 ± 147.4	587.0 ± 119.0	447.0 ± 72.1	468.3 ± 67.9	530.0 ± 207.8
*p* value	0.014	0.895	0.194	0.809	0.931	0.478	0.732	0.442

None of the patients developed severe hemorrhagic complications throughout PI-RRT. No adverse events occurred in the AN69ST group. A 5.3% incidence of adverse events occurred during the PI-RRT sessions in the AN69 group (muscle shaking in one patient and sudden low blood pressure of unknown origin in two patients).

## Discussion

RRT is of great importance in the supportive care of critically ill patients, who differ markedly from patients receiving routine hemodialysis therapy. Anticoagulants are key to preventing extracorporeal circuit thrombosis throughout the entire RRT process [1, 2]. However, anticoagulation can cause serious hemorrhagic complications in patients at high risk of bleeding. Premature circuit clotting also leads to unacceptable treatment failure. The PEI-binding AN69 membrane hemofilter described in this report is a surface-treated AN69 membrane. The second PEI-layer reduces the net negative charge of the membrane, enhances membrane polarity and lowers the repulsive force. By rinsing in unfractionated heparin saline, the membrane is primed with a heparin layer, which reduces membrane thrombogenicity [7, 8]. Previous studies have reported successful heparin-free extracorporeal circulation with the AN69ST filter for a maximum 6 h. However in the HepZero study [10, 12] reported by Laville M et al., the risk of premature clotting during 4-h hemodialysis reached more than 30% by use of a heparin-coated dialyzer, although it resulted in a higher success rate when compared with standard heparin-free hemodialysis (68.5% vs. 50.4%, respectively). Similarly in the present prospective, randomized self-controlled study, PI-RRT was completed for a duration of 543.1 ± 119.0 min in only 51.7% of patients using an AN69ST hemofilter. We should be aware that the risk of premature clotting of the extracorporeal circuit with either filter remains unsatisfactorily high. Overall, AN69ST hemofilter had no superiority over AN69 hemofilter in this study.

However, the coagulation disorders of critically ill patients are complicated besides anticoagulation strategies. Such disorders involve multiple factors associated with either clotting or bleeding. Many factors contribute to the patient’s bleeding risk and the success of extracorporeal circulation, including the disease-associated disorders such as an elevated Sequential Organ Failure Assessment score, mechanical ventilation, heart failure with fluid overload; an elevated ionized calcium concentration, an elevated platelet count and fibrinogen concentration, red cell transfusion, platelet factor 4 antibodies, concomitant therapies such as antiplatelet drugs; biocompatibility of the filter membranes, vascular access, and medical staff-related factors [18]. Our findings in the present study re-emphasize the marked influence of an elevated platelet count and hemoglobin concentration on filter clotting. Unfractionated heparin plays a role in anticoagulation by enhancing antithrombin III activity, inhibiting conversion from fibrinogen to fibrin and aggregation of red blood cells. Its anticoagulative effect is usually monitored by aPTT level. The heparin primed on the AN69ST filter membrane surface likely reduces local thrombogenesis in a similar manner. The heparin-primed AN69ST hemofilter has limited anticoagulant ability and lifespan, depending on the amount of heparin primed and the degree of heparin shedding from the membrane [19]. For patients with an increased aPTT before RRT, which indicating the occurrence of disease-related coagulation disorder, locally primed heparin will have little effect on further prolongation of the filter lifespan. In cases with severe anemia, the inhibition of RBC aggregation against fibrin formation by use of heparin, would also be weakened. Thus, when regrouping patients according to the aPTT, hemoglobin concentration and platelet count (aPTT <35.3 s, median hemoglobin concentration > 83 g/L, and platelet count <70 × 10^9^/L), we found a significant difference between the paired AN69ST and AN69 membranes, with an extra 251.7-min extension using the AN69ST hemofilter.

In fact, the result of present study does not contradict with previous reports. Another crossover study also reported that AN69ST membrane filter did not prolong filter survival during anticoagulant-free CVVHF [20]. The difference is that a majority of subjects received anti-platelet drugs (20.5%) and a daily 20-mg dose of enoxaparin (66.7%) in Schetz M’s study, thus the mean survival times of both AN69ST and AN69 hemofilters were satisfactory (14.2 and 13.3 h, respectively). Additionally, the mean platelet count was 93 × 10^9^/L (range, 52–197 × 10^9^/L), which was lower than that in the present study (126.4 ± 91.4 × 10^9^/L; range, 6–538 × 10^9^/L); notably, the platelet count was considered the strongest risk factor for filter clotting (OR, 1.07 per 10× 10^9^/L; *P* < 0.05).

Use of the original AN69 filter in RRT was historically associated with increased hypersensitivity reactions due to bradykinin release [21–23]. Coating the AN69 membrane with PEI did not alter its biocompatibility but reductions in the kallikrein activity and bradykinin concentration were observed, and no hypersensitivity reactions were reported when AN69 membranes were changed to AN69ST membranes [24–26]. This finding was confirmed in the present study. Moreover, no patient experienced a severe hemorrhagic incident.

PI-RRT serves as a transition therapy from CRRT to maintenance hemodialysis because of its shorter duration. It is being prescribed as primary therapy at a higher frequency than in the past. Marshall et al. [16] presented mortality data from three intensive care units in different countries and found that switching of the predominant therapeutic approach from CRRT to PI-RRT was not associated with any changes in the mortality rate. Basing on results of our study, AN69ST hemofilter might yet be regarded an alternative choice to RCA for PI-RRT in patients with a high bleeding risk although it is relatively inferior [27]. In sepsis, the AN69ST membrane is also recommended because of its superior cytokine-adsorptive property and ability to provide continuous 8-h therapy until the filter change.

The present study has some important limitations. Although this was a real-world study of patients undergoing PI- RRT, the number of patients in whom superiority of AN69ST was shown was limited because of the low statistical power. The pre-set washout period was not long enough for the next PI-RRT. We unfortunately did not collect time series data of aPTT during RRT, no detailed information about heparin-shedding could be provided. But in previous study, a transient increase in aPTT was observed shortly after initiation of hemodialysis, suggesting heparin shedding from the tubing and membrane [19], that might shorten the actual filter lifespan. Thus an upgrade filter grafted with heparin during manufacturing process is preferred because of its strong stability. Therefore, further studies are necessary. However heparin-coated filter is not available to clinical trial in China yet. Additionally, it is important to precisely estimate the coagulation status in an entity before decision of either anticoagulation strategies [28], and proper risk grading is needed.

## Conclusions

In conclusion, anticoagulant-free PI-RRT using a heparin-primed AN69ST hemofilter reached a success rate of 51.7%. However, the risk of premature clotting of the extracorporeal circuit remained unsatisfactorily high. The platelet count and hemoglobin concentration were strong risk factors associated with filter clotting. For select patients, the AN69ST hemofilter may be a more appropriate choice than the original AN69 filter.

## Abbreviations

Alb: Serum albumin
aPTT: Activated partial thromboplastin time
BUN: Blood urea nitrogen
CRRT: Continuous renal replacement therapy
CVVHF: Continuous venovenous hemofiltration
OR: Odds ratio
PI-RRT: Prolonged intermittent renal replacement therapy
PT: Prothrombin time
RRT: Renal replacement therapy
SCr: Serum creatinine
UF: Ultrafiltration
URR: Urea reduction ratio
